# Case report: Azithromycin-meropenem combination therapy as a low-cost approach to combat PDR gram-negative infections of war wounds in Ukraine

**DOI:** 10.3389/fmed.2023.1264492

**Published:** 2023-09-22

**Authors:** Vadym Kryzhevskyi, Viktor Strokous, Yurii Lifshyts, Yurii Rybianets, Artem Oberniak, Alexey Krikunov, Olga Iungin, Viktoria Potochilova, Kateryna Rudnieva, Pavlo Petakh, Aleksandr Kamyshnyi, Olena Moshynets

**Affiliations:** ^1^Kyiv City Clinical Hospital No 6, Kyiv, Ukraine; ^2^Oxford Medical Clinic, Kyiv, Ukraine; ^3^Bila Tserkva City Hospital No 2, Bila Tserkva, Ukraine; ^4^Shupyk National Healthcare University of Ukraine, Kyiv, Ukraine; ^5^Amosov National Institute of Cardiovascular Surgery, Kyiv, Ukraine; ^6^Biofilm Study Group, Department of Cell Regulatory Mechanisms, Institute of Molecular Biology and Genetics, National Academy of Sciences of Ukraine, Kyiv, Ukraine; ^7^Department of Biotechnology, Leather and Fur, Faculty of Chemical and Biopharmaceutical Technologies, Kyiv National University of Technologies and Design, Kyiv, Ukraine; ^8^Kyiv City Maternity Hospital No 2, Kyiv, Ukraine; ^9^Bogomolets National Medical University, Kyiv, Ukraine; ^10^Department of Microbiology, Virology, and Immunology, I. Horbachevsky Ternopil National Medical University, Ternopil, Ukraine; ^11^Department of Biochemistry and Pharmacology, Uzhhorod National University, Uzhhorod, Ukraine

**Keywords:** MDR, PDR, XDR gram-negative infections, low-cost therapy, pandrug resistance, hospital infection, war wounds, Russian-Ukrainian war

## Abstract

Antimicrobial resistance recognised as a major global health problem and it poses a significant challenge in conflict zones, such as the Russia-Ukraine war. This case study focuses on a 32-year-old soldier who sustained combat-related injuries, including extensive wound infections caused by multidrug-resistant and pan-resistant bacteria and was successfully treated with azithromycin-meropenem combination therapy. The emergence of pan-resistant bacteria, particularly a pandrug-resistant strain of *Pseudomonas aeruginosa*, highlights the severity of the problem and the limited treatment options available. Additionally, the financial burden posed by reserve antibiotics further complicates the management of these infections. The case study demonstrates the effectiveness of including azithromycin-meropenem combination therapy in the treatment regimen, which resulted in improvements in the patient’s condition and the eradication of the resistant strains. The findings underscore the need for effective antimicrobial stewardship, infection control measures, and alternative treatment strategies to combat antimicrobial resistance in conflict zones.

## Introduction

Antimicrobial resistance is one of the most serious global public health threats. Wars are often associated with antibiotic resistance outbreaks, and, consequently, the conflict in Ukraine has significantly impacted the local and global spread of аntimicrobial resistance (1–3). Combat-related injuries often present complex and challenging medical scenarios that require rapid and comprehensive treatment to ensure the best possible outcomes. In the context of conflict, such as the Russia-Ukraine war, healthcare systems face unique challenges (4–7). Consequently, the Russian war against Ukraine dramatically influenced on antibiotic resistance local and global spread (8–12).

One of the alarming consequences of the Russia-Ukraine war is the emergence of super-resistant bacteria, or superbugs, including pandrug-resistant (PDR) strains. Pan-resistant bacteria are resistant to all available antibiotics, leaving healthcare providers without standard treatment options. This not only possess a significant threat to the lives of war-wounded patients but also presents a challenge in terms of infection control and public health (12, 13).

Furthermore, the high cost of reserve antibiotics further exacerbates the challenges faced by healthcare providers (12, 14). In 2021, the WHO updated the AWaRe classification of antibiotics; this included eight reserve antibiotics for the treatment of multidrug-resistant pathogens. It contains cefiderocol, ceftazidime + avibactam, colistin, fosfomycin, linezolid, meropenem + vaborbactam, plazomicin, and polymyxin B (15) (cited July 7, 2023). However, their limited availability and high prices hinder their accessibility, particularly in resource-constrained conflict settings. This scarcity of effective antibiotics adds another layer of complexity to the management of combat-related injuries, jeopardizing the already precarious health outcomes for patients.

This case report focuses on the treatment of a 32-year-old soldier who sustained substantial injuries during battle in Ukraine. By examining the patterns of antimicrobial resistance observed in a war-wounded patient from Ukraine, this case study aims to shed light on the difficulties associated with pan-resistant bacteria in the context of conflict and suggests a new therapeutic approach that may be applied when PDR Gram-negative infection. Following the CARE checklist, this report aims to provide a thorough analysis of the case, ensuring transparency and adherence to established reporting guidelines (https://www.care-statement.org/, accessed on July 15, 2023).

### Patient information

A 32-year-old male soldier received a penetrating abdominal injury after a fight in the Donbas. Initial medical care was provided by the advanced surgical group of the reinforcement in Druzkivka; following stabilization the patient was transferred to the Dnipro Regional Clinical Hospital by I. I. Mechnikov the same day. Upon admission, the patient’s condition was critical. He was under medical sedation and had a plaster cast on his left hand. Breathing assistance was provided through an endotracheal tube connected to a transport ventilator, initially through a Maquet respirator in Synchronized Intermittent Mandatory Ventilation (SIMV) mode. Hemodynamics were stable with normal blood pressure. Heart sounds were muffled. The patient’s abdomen was tense, and no peristalsis was audible. Abdominal bandages showed saturation with blood. Urinary output was managed through a Foley urinary catheter, and central venous catheters had been inserted in the right and left subclavian vein.

### Clinical findings

The patient presented with a series of significant clinical findings:

1. Gunshot shrapnel blind penetrating abdominal injury with damage to the small and large intestine.
2. Hemoperitoneum, indicating the presence of blood in the peritoneal cavity.
3. Entrapment of the small intestine, complicating the patient’s condition.
4. Significant soft tissue defect of the anterior abdominal wall.
5. Gunshot fracture of the 3rd finger at the level of the middle phalanx.
6. Open fracture of the 2nd and 5th fingers at the level of the distal phalanx of the left hand with a foreign body.

### Timeline

On April 20, 2023, the patient was admitted to the Dnipro Regional Clinical Hospital by I. I. Mechnikov. A laparotomy was performed, and the damaged small intestine was sutured. The colon underwent obstructive resection, and an abdominal tamponade was applied as part of damage control surgery. Additionally, the patient received primary surgical treatment for the left-hand wound, including removal of a foreign body and application of a plaster cast.

The following day, April 21, 2023, a relaparotomy was conducted, along with an abdominal cavity revision. The small intestine and descending colon were resected, and a side-to-side entero-enteroanastomosis was performed. An ileostomy was created, and abdominal tamponade was implemented.

On April 24, 2023, another relaparotomy was carried out to inspect the abdominal cavity. A descending-sigmoid anastomosis was performed, and the large cap was resected. Tampons were removed, and a necrectomy of the anterior abdominal wall wound was performed. A Vacuum-Assisted Closure (VAC) system was installed to aid in wound healing.

A lower tracheostomy was performed on April 26, 2023, followed by a removal of the VAC system on April 27, 2023. A relaparotomy was performed to revise the abdominal cavity, suture the small intestine defect, and reinstall the VAC system.

The patient stayed in the Dnipro Regional Clinical Hospital by I. I. Mechnikov for eight days before he was transferred to Kyiv City Clinical Hospital No 6 in Kyiv. During these eight days, there was no microbiological investigation performed with, however, empirical antibiotic therapy with ceftazidim (20–23 of April), meropenem (23–28 of April), linezolid (20–28 of April), metronidazole (20–28 of April), and clindamycin (25–28 of April).

### Diagnostic assessment

Upon arrival at Kyiv City Clinical Hospital No 6 the diagnostic assessments performed on the patient included a CT scan of the chest and abdominal cavities, a clinical blood count, leukocyte count, and measurement of procalcitonin levels. The CT scan revealed multiple fragments in the abdominal cavity. The clinical blood count assessed various blood cell counts. Procalcitonin levels were measured as a biomarker for bacterial infections.

Microbiology analyses were carried out on sputum, wound, urine samples and blood catheters regularly to monitor microbial infections. The samples underwent laboratory testing to determine the specific microorganisms present and assess their susceptibility to a range of antibiotics. The antibiotic susceptibility testing was performed using the disk-diffusion method in accordance with the guidelines set by the European Committee on Antimicrobial Susceptibility Testing (EUCAST) (Version 13.0, 2023, for all of the antibiotics except tigecycline where Version 8.0, 2018, was used. http://www.eucast.org, accessed on July 15, 2023).

The wound samples obtained from the patient revealed the presence of multidrug-resistant (MDR) strains of *Escherichia coli* and *Klebsiella pneumoniae*. Furthermore, extensively drug-resistant (XDR) strains of *E. coli*, *K. pneumoniae* and *Acinetobacter baumannii* were also isolated from the wound. Notably, a pandrug resistant (PDR) strain of *P. aeruginosa* was identified, indicating resistance to a wide range of antimicrobial agents.

Similarly, the sputum samples (due to a ventilation associated pneumonia, VAP) collected from the patient exhibited the presence of MDR strains of *E. coli* and *A. baumannii*. Additionally, a PDR strain of *P. aeruginosa*, as well as XDR strains of *K. pneumoniae* and *A. baumannii* were isolated from the sputum (Supplementary material).

### Therapeutic intervention

**Figure 1:**
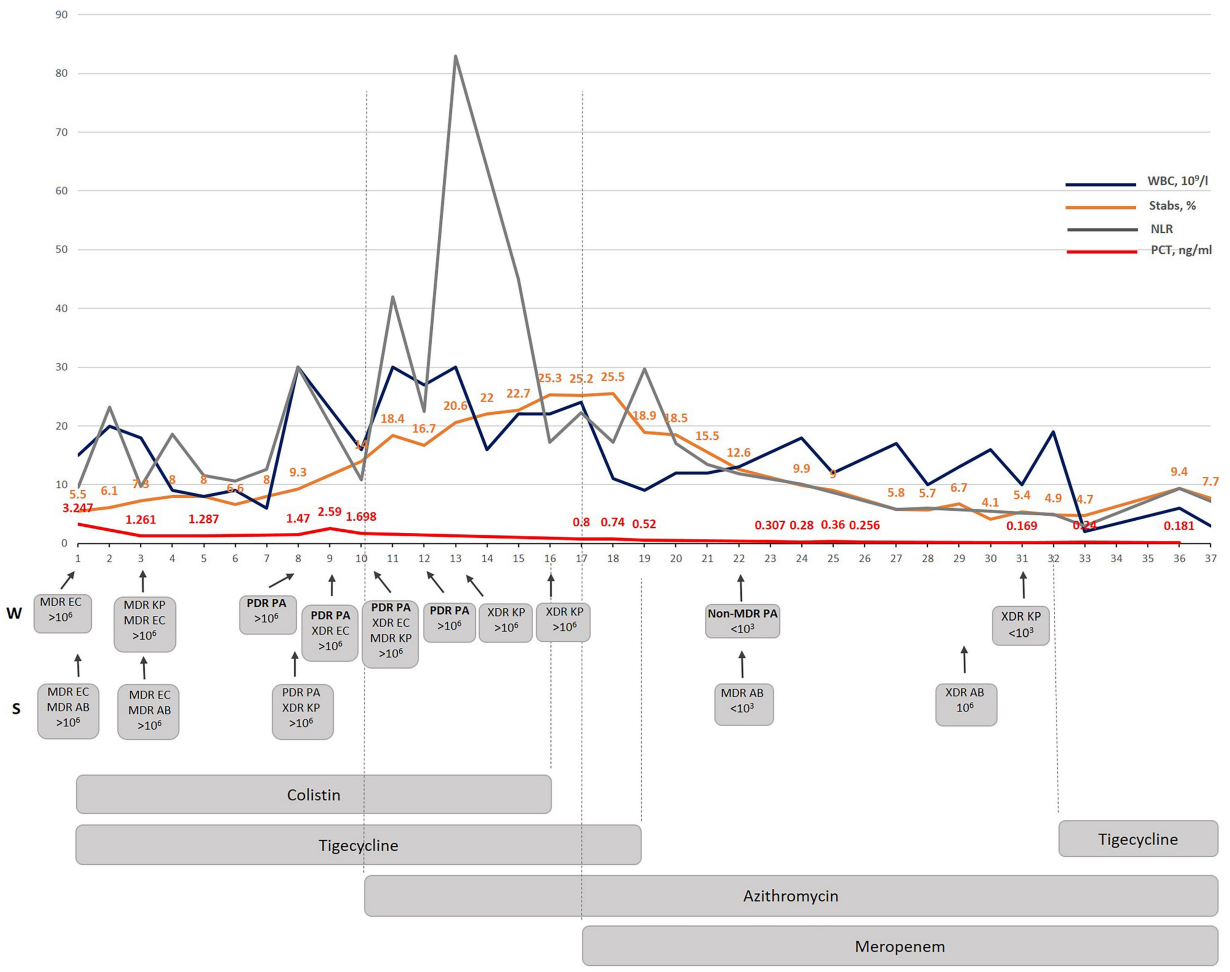
The dynamic correlation of some inflammatory markers as well as WBC and stabs during antibacterial treatment performed in Kyiv City Clinical Hospital No 6. WBC-white blood cells; NLR- a neutrophil lymphocyte ratio; PCT – procalcitonin; W-wound; S – sputum; MDR – multidrug-resistant; XDR – extensively drug-resistant; PDR – pandrug resistant; EC – *E. coli*; AB – *A. baumannii*; KP – K. *pneumoniae*; PA – *P. aeruginosa*.

The following medications were administered during treatment in Kyiv City Clinical Hospital No 6 (Figure 1): sodium colistimethate (colistin) was prescribed at a dosage of 2 MO every 8 h IV from the 1st to the 16th day of treatment. 50 mg tigecycline was given IV every 12 h from day 1 to 19 and from day 32 to 31. Azithromycin, a macrolide antibiotic, was included in the treatment plan. The patient received a dosage of 500 mg of azithromycin every 8 h IV from the 10th to the 37th day. Meropenem, a carbapenem antibiotic, was prescribed at a dosage of 2 g every 8 h IV from the 17th to the 37th day of treatment.

### Outcomes

The introduction of azithromycin into the treatment regimen on day 10 resulted in notable improvements in the patient’s condition. Procalcitonin level, which serve as a marker for bacterial infection, showed a consistent decline over the course of treatment (Figure 1). On day 10, the procalcitonin level was measured at 1.69 ng/mL. Within 7 days (by day 17) it decreased to 0.8 ng/mL during which the same antibiotics were given as since the beginning of the therapy (colistin and tigecycline). By day 23 it had decreased to 0.307 ng/mL, and further decreased to 0.18 ng/mL by day 36.

The appearance of a PDR *P. aeruginosa* isolate correlated with the increase in WBC and NLR indicating a considerable immunogenicity of the *P. aeruginosa* isolate found the same day in both wound and sputum samples. A significant NLR increase occurred three days from the moment when PDR *P. aeruginosa* was detected, and azithromycin therapy was started. A considerable dynamic reduction in stabs and PCT started on the 7th day when both colistin and tigecycline were replaced with meropenem even though all of the detected isolates were resistant to this antibiotic. Interestingly, the PDR *P. aeruginosa* isolate was replaced with a non-MDR *P. aeruginosa* isolate sensitive to five antibiotics which were ineffective against the previous *P. aeruginosa* isolate (Supplementary material). The meropenem added on the 7th day since the azithromycin therapy started (day 17th of the treatment in Kyiv), considerably improved PCT, WBC, stabs dynamics and resulted in a complete normalization by day 36. The subsequent monitoring and treatment strategy led to the complete eradication of the PDR/non-MDR *P. aeruginosa* strains by day 31. This successful outcome demonstrates the effectiveness of the implemented therapeutic interventions in targeting and eliminating the PDR *P. aeruginosa* -associated infection.

**Figure 2:**
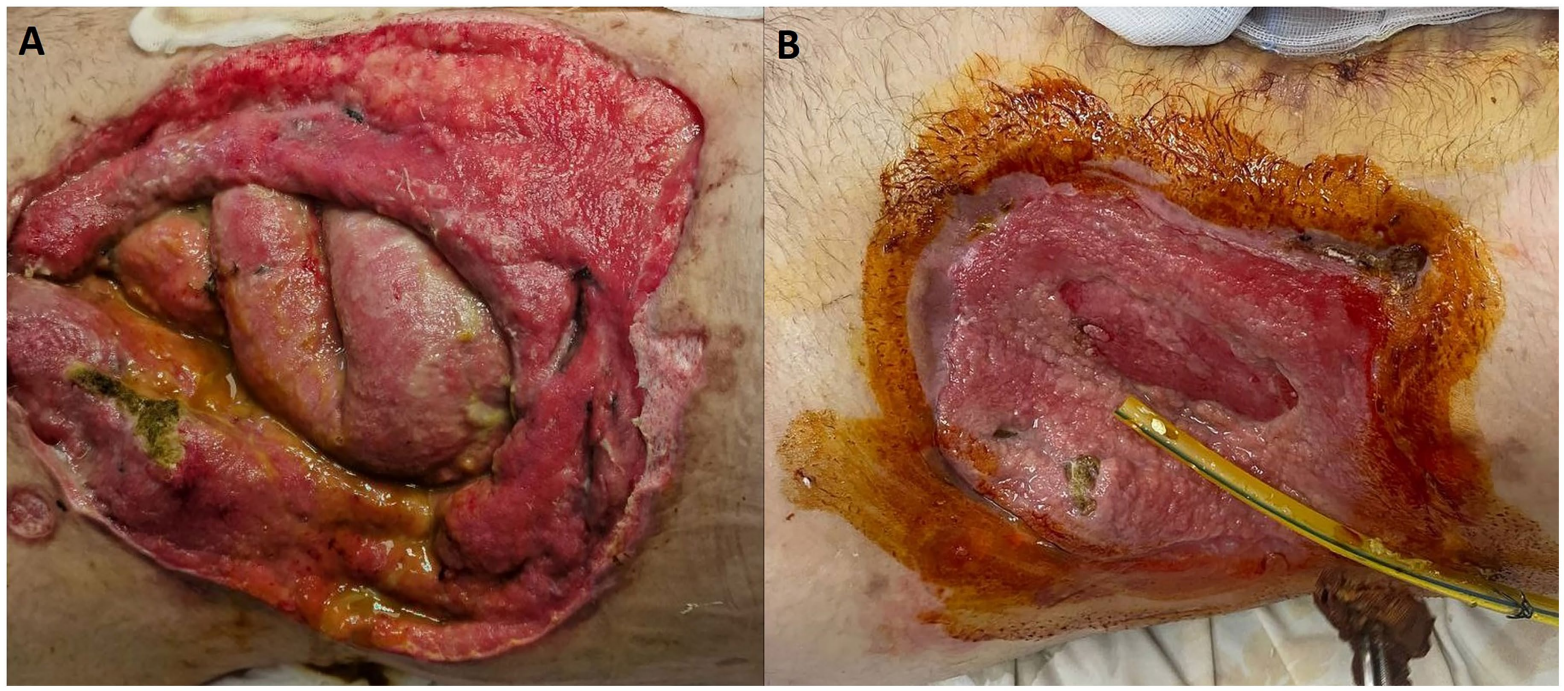
The 9-day old **(A)** and the same wound on day 36 **(B)**.

Furthermore, encouraging progress was observed in the healing of the stoma wound. The wound underwent a process of granulation, which involves the formation of new tissue, and exhibited signs of healing. This positive development suggests that the combination of the prescribed medications and wound care measures facilitated the healing process (Figure 2).

Systemic antimicrobial therapy was stopped on the 37th day, when the patient was stable, with no recurrence of the sepsis markers and a PCT not higher that 0.25 ng/mL for the following two weeks and he finally was released from the intensive care unit.

## Discussion

**Figure 3:**
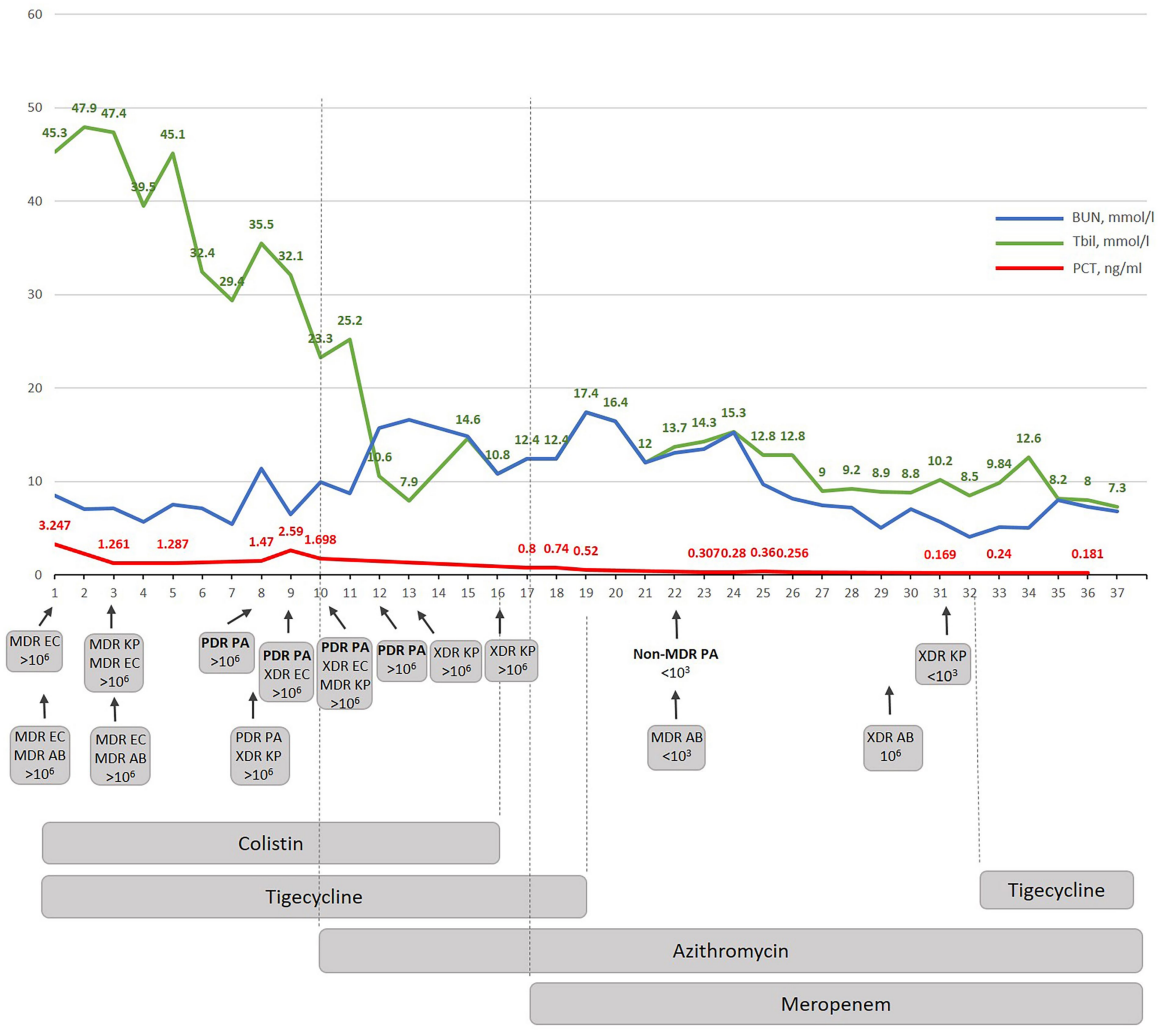
The dynamic correlation of some biochemical markers during antibacterial treatment performed in Kyiv City Clinical Hospital No 6. BUN-blood urea nitrogen; Tbil-total bilirubin; PCT – procalcitonin; W-wound; S – sputum; MDR – multidrug-resistant; XDR – extensively drug-resistant; PDR – pandrug resistant; EC – *E. coli*; AB – *A. baumannii*; KP – *K. pneumoniae*; PA – *P. aeruginosa*.

Sepsis caused by super-resistant multi-infection was diagnosed in the patient. Until transportation to Kyiv City Clinical Hospital No 6 and within the first 8 days, only MDR gram-negative isolates were detected. It is important to emphasize that all these four isolates were sensitive to tigecycline, and they hypothetically may be still sensitive to colistin, as colistin-resistance is still not so often encountered in Ukrainian hospital isolates, as demonstrated before (16) or as demonstrated by other authors (17). However, despite this rational combined antimicrobial therapy with colistin and tigecycline, the condition of the patient decreased: he had hypothermia, anemia, underwent two blood transfusions, his bilirubin level was increased and PCT varied from 3.247 to 1.698 ng/mL (Figure 3). All this indicated that phenotypic resistance to both colistin and tigecycline developed following biofilm formation during infection process despite the confirmed genetic sensitivity to one antibiotic and expected sensitivity to another. The phenotypic resistance should always be suspected if a recommended rational therapy does not improve the condition of a patients within three days.

The newly suggested therapy was focused on anti-biofilm therapy and included azithromycin which was effective against gram-negative XDR/PDR Ukrainian Hospital Isolates as shown before (16, 18). The combination therapy with azithromycin and meropenem was suggested based on own previous experimental and clinical data which confirmed that both antibiotics demonstrated a strong synergistic effect *in vivo* against diverse gram-negative hospital isolates. Moreover, this synergism cannot be observed for individual isolates in the standard recommended *in vitro* assays as it is biofilm dependent and specific *in vivo* due to enhanced bactericidal efficacy of AZM in a physiologically relevant environment. In other words, when the infection is caused by gram-negative bacteria with the XDR/PDR phenotype, azithromycin-meropenem can be used without additional synergism testing. Indeed, the beginning of the azithromycin therapy provoked immunostimulation (quick rise of NLR and stabs) but a decrease in PCT (0.8 ng/mL) (Figure 1). Since colistin therapy demonstrated nephrotoxicity (BUN increased up to 18 mmoL/L) (Figure 3) and because our new experimental data demonstrates that the adaptation to azithromycin mitigates PDR phenotype and restores sensitivity to meropenem in PDR *K. pneumoniae* (Abstract No 03094, Poster No P04461 presented as a poster during ECCMID 2023) colistin and tigecycline were replaced with meropenem (19). This resulted in fast normalization of PCT (from 0.8 to less than 0.25 ng/mL), normalization of WBS, stabs and NLR. Interestingly, as predicted in our research, PDR *P. aeruginosa* restores the sensitivity to meropenem, but not only to that. In general, PDR *P. aeruginosa* was replaced with non-MDR *P. aeruginosa* sensitive to five antibiotics on day 12 of azithromycin therapy and following 5 days of therapy with meropenem (Supplementary material). 1,500 mg of azithromycin daily was nicely tolerated; the only observed side effect was the appearance of individual myelocytes and blast cells in peripheral blood which might be explained by the inhibition of nuclear factor kappa B (NF-kB) by azithromycin (20) which it turn promotes myelogenesis (21). Atypical cells disappeared within three days after completion of the azithromycin therapy.

The successful use of azithromycin-meropenem combination demonstrates a low-cost approach for combating XDR/PDR in multiple gram-negative infections of war wounds in Ukraine. Another approach such as cefiderocol is unlikely to be effective in Ukraine due to its high price and fast resistance development (12). Berger (12) also suggests to use a combination of ceftazidime/avibactam and aztreonam, as this would restore the sensitivity to NDM-producing gram-negative hospital isolates. The latter are present in up to 99% of the Ukrainian hospital isolates (even carbapenem-sensitive, unpublished data), However, this may not be effective in Ukraine due to the low quality of the available aztreonam (personal observation).

## Data availability statement

The raw data supporting the conclusions of this article will be made available by the authors, without undue reservation.

## Ethics statement

The studies involving humans were approved by Institute of Molecular Biology and Genetics. The studies were conducted in accordance with the local legislation and institutional requirements. The participants provided their written informed consent to participate in this study. Written informed consent was obtained from the individual(s) for the publication of any potentially identifiable images or data included in this article. Written informed consent was obtained from the participant/patient(s) for the publication of this case report.

## Author contributions

KV: Formal analysis, Data curation, Methodology, Resources. SV: Data curation, Formal analysis, Methodology, Resources, Visualization. LY: Data curation, Formal analysis, Methodology, Resources. RY: Data curation, Formal analysis, Methodology, Resources. OA: Formal analysis, Methodology. KrA: Conceptualization, Investigation, Methodology. IO: Investigation, Methodology. PV: Investigation, Methodology. RK: Investigation, Methodology. PP: Formal analysis, Writing – original draft, Writing – review & editing. KaA: Formal analysis, Conceptualization, Writing – original draft, Writing – review & editing. OM: Data curation, Formal analysis, Funding acquisition, Investigation, Methodology, Project administration, Resources, Supervision, Validation, Visualization, Writing – original draft, Writing – review & editing.

## Funding

The author(s) declare financial support was received for the research, authorship, and/or publication of this article. This research was funded by the National Research Foundation of Ukraine (https://nrfu.Org.ua/en/) (accessed on 17–18 September 2020, protocol No 21) within the frame of the project “Development of combined therapy for severe Klebsiella pneumoniae–associated nosocomial infections to overcome the antibiotic resistance” (2020.02/0246).

## Conflict of interest

The authors declare that the research was conducted in the absence of any commercial or financial relationships that could be construed as a potential conflict of interest.

## Publisher’s note

All claims expressed in this article are solely those of the authors and do not necessarily represent those of their affiliated organizations, or those of the publisher, the editors and the reviewers. Any product that may be evaluated in this article, or claim that may be made by its manufacturer, is not guaranteed or endorsed by the publisher.

## References

[1] Älgå A Karlow Herzog K Alrawashdeh M Wong S Khankeh H Stålsby LC. Perceptions of healthcare-associated infection and antibiotic resistance among physicians treating Syrian patients with war-related injuries. Int J Environ Res Public Health. (2018) 15:2709. doi: 10.3390/ijerph15122709, PMID: 30513739 PMC6313556

[2] Jakovljevic M Al Ahdab S Jurisevic M Mouselli S. Antibiotic resistance in Syria: a local problem turns into a global threat. Front Public Health. (2018) 6:212. doi: 10.3389/fpubh.2018.00212, PMID: 30116726 PMC6084506

[3] Yaacoub S Truppa C Pedersen TI Abdo H Rossi R. Antibiotic resistance among bacteria isolated from war-wounded patients at the weapon traumatology training Center of the International Committee of the red cross from 2016 to 2019: a secondary analysis of WHONET surveillance data. BMC Infect Dis. (2022) 22:257. doi: 10.1186/s12879-022-07253-1, PMID: 35287597 PMC8922823

[4] Kardas P Babicki M Krawczyk J Mastalerz-Migas A. War in Ukraine and the challenges it brings to the polish healthcare system. Lancet Reg Health Eur. (2022) 15:100365. doi: 10.1016/j.lanepe.2022.100365, PMID: 35531498 PMC9073000

[5] Shkodina AD Chopra H Singh I Ahmad S Boiko DI. Healthcare system amidst the war in Ukraine. Ann Med Surg (Lond). (2022) 80:104271. doi: 10.1016/j.amsu.2022.104271 35958284 PMC9358411

[6] Petakh P Kamyshnyi A. Risks of outbreaks: the health concerns of internally displaced persons in Transcarpathia, Ukraine. New Microbes New Infect. (2023) 52:101106. doi: 10.1016/j.nmni.2023.101106, PMID: 36874153 PMC9982595

[7] Petakh P Kamyshnyi A Tymchyk V Armitage R. Infectious diseases during the Russian-Ukrainian war – morbidity in the Transcarpathian region as a marker of epidemic danger on the EU border. Public Health Pract. (2023) 6:100397. doi: 10.1016/j.puhip.2023.100397, PMID: 37449295 PMC10336168

[8] Kondratiuk V Jones BT Kovalchuk V Kovalenko I Ganiuk V Kondratiuk O. Phenotypic and genotypic characterization of antibiotic resistance in military hospital-associated bacteria from war injuries in the eastern Ukraine conflict between 2014 and 2020. J Hosp Infect. (2021) 112:69–76. doi: 10.1016/j.jhin.2021.03.020, PMID: 33789157

[9] Melwani M. How war is spreading drug resistant superbugs across Ukraine and beyond. BMJ. (2022) 379:o2731. doi: 10.1136/bmj.o2731 36414262

[10] Murray CJL Ikuta KS Sharara F Swetschinski L Robles Aguilar G Gray A. Global burden of bacterial antimicrobial resistance in 2019: a systematic analysis. Lancet. (2022) 399:629–55. doi: 10.1016/S0140-6736(21)02724-0, PMID: 35065702 PMC8841637

[11] Organization WHO. WHO response to the Ukraine crisis: March 2023 bulletin. Regional Office for Europe: World Health Organization (2023).

[12] Berger FK Schmartz GP Fritz T Veith N Alhussein F Roth S. Occurrence, resistance patterns, and management of carbapenemase-producing bacteria in war-wounded refugees from Ukraine. Int J Infect Dis. (2023) 132:89–92. doi: 10.1016/j.ijid.2023.04.394, PMID: 37072055

[13] Magiorakos AP Srinivasan A Carey RB Carmeli Y Falagas ME Giske CG. Multidrug-resistant, extensively drug-resistant and pandrug-resistant bacteria: an international expert proposal for interim standard definitions for acquired resistance. Clin Microbiol Infect. (2012) 18:268–81. doi: 10.1111/j.1469-0691.2011.03570.x, PMID: 21793988

[14] Majumder MAA Rahman S Cohall D Bharatha A Singh K Haque M. Antimicrobial stewardship: fighting antimicrobial resistance and protecting global public health. Infect Drug Resist. (2020) 13:4713–38. doi: 10.2147/IDR.S290835, PMID: 33402841 PMC7778387

[15] AWaRe classification (2021). Geneva: World Health Organization. Available at: https://www.who.int/publications/i/item/2021-aware-classification

[16] Moshynets OV Baranovskyi TP Cameron S Iungin OS Pokholenko I Jerdan R. Azithromycin possesses biofilm–inhibitory activity and potentiates non-bactericidal colistin methanesulfonate (CMS) and polymyxin B against Klebsiella pneumonia. PLoS One. (2022) 17:e0270983. doi: 10.1371/journal.pone.0270983, PMID: 35776759 PMC9249213

[17] Mc Gann PT Lebreton F Jones BT Dao HD Martin MJ Nelson MJ. Six extensively drug-resistant bacteria in an injured soldier, Ukraine. Emerg Infect Dis. (2023) 29:1692–5. doi: 10.3201/eid2908.230567, PMID: 37406356 PMC10370857

[18] Moshynets OV Baranovskyi TP Iungin OS Krikunov AA Potochilova VV Rudnieva KL. Therapeutic potential of an azithromycin-colistin combination against XDR *K. pneumoniae* in a 3D collagen-based in vitro wound model of a biofilm infection. Antibiotics (Basel). (2023) 12:293. doi: 10.3390/antibiotics12020293 36830203 PMC9952533

[19] Moshynets O Baranovskyi T Iungin O Pokholenko I Potochilova V Krikunov A. Adaptation to azithromycin mitigates PDR phenotype and restores sensitivity to meropenem in PDR Klebsiella pneumonia. 33rd. Copenhagen, Denmark: ECCMID (2023) Abstract No 03094, Poster No P0461.

[20] Iwamoto S Kumamoto T Azuma E Hirayama M Ito M Amano K. The effect of azithromycin on the maturation and function of murine bone marrow-derived dendritic cells. Clin Exp Immunol. (2011) 166:385–92. doi: 10.1111/j.1365-2249.2011.04480.x, PMID: 22059997 PMC3232387

[21] Reikvam H. Inhibition of NF-κB signaling alters acute myelogenous leukemia cell transcriptomics. Cells. (2020) 9:1677. doi: 10.3390/cells9071677, PMID: 32664684 PMC7408594

