# Supplementary material for: Case report: Azithromycin-meropenem combination therapy as a low-cost approach to combat PDR gram-negative infections of war wounds in Ukraine
Source: Front Med (Lausanne). 2023 Sep 22;10:1264492. doi: 10.3389/fmed.2023.1264492 (PMC10566362; doi:10.3389/fmed.2023.1264492)
Supplement: Supplementary file 1 [file Table_1.docx]

Supplementary Material

Azithromycin-Meropenem Combining Therapy as a Low-Cost Approach for Combating Pandrug Resistance in Multiple Gram-Negative Infections of War Wounds in Ukraine: A Case Report

**Kryzhevskyi V.^1^, Strokous V. ^1^, Lifshyts Y. ^2^, Rybianets Yu. ^1^, Oberniak A. ^3,4^, Krikunov A.^5^, Iungin O. ^6,7^, Potochilova V.^8^, Rudnieva K.^9^, Petakh P.^10,11^, Kamyshnyi O.^10^, Moshynets O.^6^***

^1^Kyiv City Clinical Hospital No 6, 3 Lubomyra Huzara Str, Kyiv 03065, Ukraine;

^2^Oxford Medical Clinic, 30b Berezniakivska Str., Kyiv 02152, Ukraine;

^3^Bila Tserkva City Hospital №2, 9 hor Zynycha Str., Bila Tserkva 09119, Ukraine;

^4^Shupyk National Healthcare University of Ukraine, Kyiv, Ukraine;

^5^Amosov National Institute of Cardiovascular Surgery, 6 N. Amosov Str, Kyiv 03038, Ukraine;

^6^Biofilm Study Group, Department of Cell Regulatory Mechanisms, Institute of Molecular Biology and Genetics, National Academy of Sciences of Ukraine, 150 Zabolotnoho Str., Kyiv 03680, Ukraine;;

^7^Department of Biotechnology, Leather and Fur, Faculty of Chemical and Biopharmaceutical Technologies, Kyiv National University of Technologies and Design, Mala Shyianovska Street, 2, Kyiv 01011, Ukraine;

^8^Kyiv City Maternity Hospital № 2, Mostytska 11, Kyiv 02000, Ukraine;

^9^Bogomolets National Medical University, 13 Shevchenka Blvd, Kyiv 01601, Ukraine;

^10^Department of Microbiology, Virology, and Immunology, I. Horbachevsky Ternopil National Medical University, Ternopil 46001, Ukraine;

^11^Department of Biochemistry and Pharmacology, Uzhhorod National University, 88000 Uzhhorod, Ukraine;

*** Correspondence:** moshynets@gmail.com

## Supplementary Table 1. Origin and antibiotic sensitivities of the isolates.

| **Day of hospital stay** | **1,3** | **1,3** | **3** | **8** | **8** | **9,10,12,16** | **9,10** | **10** | **13** | **22** | **22** | **24** | **29** | **31** |
| --- | --- | --- | --- | --- | --- | --- | --- | --- | --- | --- | --- | --- | --- | --- |
| **Sample origin^1^** | **W,S** | **S** | **W** | **W** | **W,S** | **W** | **W** | **W** | **W** | **S** | **W** | **C** | **S** | **W** |
| **Isolate^2^** | EC | AB | KP | KP | PA | PA | EC | KP | KP | AB | PA | KP | AB | KP |
| **Contamination level, CFU** | >10^6^ | >10^6^ | >10^6^ | >10^6^ | >10^6^ | >10^6^ | >10^6^ | >10^6^ | >10^6^ | <10^3^ | <10^3^ | <10^3^ | >10^6^ | <10^3^ |
| **Phenotype characteristics^3^** | MDR | MDR | XDR | MDR | **PDR** | **PDR** | XDR | MDR | MDR | MDR | **Non-MDR** | MDR | XDR |  |
| **Antibiotic sensitivity**^4^ | | | | | | | | | | | | | | |
| Azlocillin | R | R | R | R | **R** | **R** | R | R | R | R | **R** | R | R | R |
| Cefepime | R | R | R | R | **R** | **R** | R | R | R | R | **I** | R | R | R |
| Cefoperazone/sulbactam | R | S | I | R | **R** | **R** | I | R | R | S | **S** | R | NA | NA |
| Cefotaxime | R | R | NA | R | **NA** | **NA** | R | R | R | NA | **I** | R | R | R |
| Ceftazidime | R | R | R | R | **R** | **R** | R | R | R | R | **NA** | R | R | R |
| Ceftazidime/avibactam | S | R | R | R | **R** | **R** | R | R | NA | NA | **NA** | NA | R | NA |
| Ceftriaxone | R | NA | R | R | **NA** | **NA** | R | NA | R | NA | **NA** | R | R | R |
| Amykacin | S | R | R | S | **R** | **R** | I | S | S | R | **S** | S | NA | NA |
| Gentamicin | S | NA | NA | NA | **NA** | **NA** | NA | NA | S | NA | **NA** | NA | NA | NA |
| Tobramycin | NA | R | S | I | **R** | **R** | R | R | I | R | **R** | I | R | S |
| Piperacillin | R | R | R | R | **NA** | **NA** | NA | NA | R | R | **S** | R | R | R |
| Norfloxacin | R | R | R | R | **R** | **R** | R | R | R | R | **R** | NA | R | NA |
| Ciprofloxacin | R | R | R | R | **R** | **R** | R | R | R | R | **R** | R | R | R |
| Levofloxacin | R | R | R | R | **R** | **R** | R | R | R | R | **R** | R | R | R |
| Doripenem | R | R | R | R | **R** | **R** | NA | NA | R | R | **S** | R | R | R |
| Meropenem | R | NA | NA | R | **R** | **R** | NA | NA | R | R | **S** | R | R | R |
| Aztreonam | NA | NA | NA | NA | **NA** | **R** | NA | NA | R | NA | **NA** | NA | NA | NA |
| Tigecycline | S | S | I | S | **R^5^** | **R^5^** | S | S | R | S | **R^5^** | S | I | I |
| Colistin^6^ | NA | NA | NA | NA | **NA** | **NA** | NA | NA | NA | NA | **NA** | NA | NA | NA |
| ^1^ Isolates were recovered from a wound (W), sputum (S) and a subclavian catheter (C); ^2^ EC corresponds to *Escherichia coli*, AB corresponds to *Acinetobacter baumannii*, KP corresponds to *Klebsiella pneumonia* and PA corresponds *Pseudomonas aeruginosa*; ^3^ Strain considered as MDR when it is sensitive to two or more antibiotics, XDR phenotype corresponded to sensitivity to only one antibiotics, PDR phenotype corresponded to full resistance; ^4^ Antibiotic susceptibilities determined from disc-diffusion assays according to "The European Committee on Antimicrobial Susceptibility Testing. Breakpoint tables for interpretation of MICs and zone diameters. Version 13.0, 2023. http://www.eucast.org." except for tigecycline where Version 8.0, 2018 was used and are shown as sensitive (S), intermediate (I) or resistant (R); ^5^ PA has a natural resistance to tigecycline ^6^ Isolates were not tested against colistin due to unavailability of a recommended test system. | | | | | | | | | | | | | | |
